# Acylation of Lignin with Different Acylating Agents by Mechanical Activation-Assisted Solid Phase Synthesis: Preparation and Properties

**DOI:** 10.3390/polym10080907

**Published:** 2018-08-12

**Authors:** Xiaohong Zhao, Yanjuan Zhang, Mei Yang, Zuqiang Huang, Huayu Hu, Aimin Huang, Zhenfei Feng

**Affiliations:** 1 School of Chemistry and Chemical Engineering, Guangxi University, Nanning 530004, China; mshong_200@163.com (X.Z.); yangmei@gxu.edu.cn (M.Y.); yuhuahu@163.com (H.H.); ham2003cn@163.com (A.H.); myAfeila@163.com (Z.F.); 2 College of Materials and Environmental Engineering, Hezhou University, Hezhou 542899, China

**Keywords:** lignin, acylation, mechanical activation, solid phase synthesis

## Abstract

Acylated lignins with substituents consisting of different lengths of carbon chains were prepared by a mechanical activation-assisted solid phase synthesis (MASPS) technology with a customized stirring ball mill as a reactor. The structures and properties were analyzed by UV/Vis, FTIR, NMR, SEM, DSC, and TG. The results showed that the acylated lignins were successfully prepared with either non-cyclic or cyclic anhydrides as the acylating agents. Both aliphatic hydroxyl and phenolic hydroxyl groups of lignin reacted with non-cyclic anhydrides, and different reactivity of acylating agents resulted in different relative contents of phenolic and aliphatic substituents in the products. The reactivity of the cyclic anhydrides was weaker than that of the non-cyclic anhydrides, and the reactivity of the acylating agents decreased with increasing carbon chain length and unsaturated bonds of acyl groups. All of the acylated lignins except maleylated lignin had a lower glass transition temperature (*T*_g_) than the original lignin. The acylated lignins prepared with non-cyclic anhydrides had better thermal stability than original lignin, and the thermal stability increased, but *T*_g_ decreased with an increasing chain length of the acyl groups. The acylated lignins prepared with cyclic anhydrides had higher a *T*_g_ than those with non-cyclic anhydrides with the same carbon number, and the thermal stability was not obviously improved.

## 1. Introduction

Lignin is a cross-linked amorphous copolymer consisting of three phenylpropane monomers (p-coumaryl, coniferyl, and sinapyl alcohols), which are bonded together through C–O–C and C–C interunit linkages [1,2]. It is one of the main ingredients of lignocellulose, accounting for 15–25% of the total weight, and it serves as a potential source of aromatic platform chemicals, although such a conversion is still challenging [3]. With the development of the pulp and paper industry and bioethanol industry, more than 300 billion tons of lignin are available in the world, but high-value applications of lignin amount to no more than 2% [4,5]. Most of the lignin has not been efficiently used due to its complicated interunit linkages, high affinity, and the ease of forming a more condensed structure, poor product selectivity, poor compatibility with polymers, and so on [4]. Thus, studies on the exploitation and application of lignin-based products have great significance.

Modification can greatly expand the application of lignin in polymer materials and chemical syntheses [1], and esterification is an important method for the modification of lignin. The photo-thermal stability, thermal properties (e.g., molar mass, solubility, and softening temperature), compatibility with non-polar polymers, and solubility in non-polar solvents can be improved after esterification [6,7,8]. The properties of the resulting products can be tailored for various applications, such as being used as fillers, thermal stabilizers, plasticizers, and mold lubricants in composites, thermoplastic starch, cellulose triacetate films, and so on [9,10,11]. Esterified lignin was found to lead to less deterioration of mechanical properties than alkylated lignin [12]. Cyclic anhydride-modified lignin can introduce an active COOH, which makes it easy to form esters or hydrogen bonding with –OH, so that they can be used as lubricants, precursors for the synthesis of durable materials, unsaturated thermosetting materials, or biological carbon [13,14].

The esterification of lignin usually uses acid anhydride or acyl chloride as an esterifying agent, pyridine, dimethylaminopyridine (DMAP), 1-or *N*-methylimidazole (1MIM), 2-methylimidazole (2MIM), or imidazole (IM) as a catalyst, and excessive acid anhydride, pyridine, tetrahydrofuran, 1,4-dioxane, or *N*-methyl pyrrolidone as a solvent [7,10,15]. The reaction usually requires a long time, and the recovery of modified lignin is time-consuming and tedious. So, close attention has been paid to some new preparation methods: taking the supercritical carbon dioxide as a solvent can overcome the trivial recycling of the product [14]; the assistance of microwaves or ionic liquids can greatly improve the reaction rate [16,17]; reactive extrusion methods, without the use of a solvent, are green and economical [18].

Different esterifying agents and preparation methods may result in different reaction characteristics and product properties. The reactivity of phenolic hydroxyl groups is three times of that of aliphatic hydroxyl groups when the esterification is carried out in the liquid phase with a non-cyclic anhydride (acetic, propionic, butyric, or methacrylic anhydride) as an acylating agent, while the acylation of aliphatic hydroxyl is a priority when the esterification is carried out with a cyclic anhydride as an acylating agent [7,16,19]. Esterification with non-cyclic anhydrides has a higher degree of substitution (DS) than esterification with cyclic anhydrides, and DS decreases with increasing carbon chain length, and increase in the number of unsaturated bonds of aliphatic anhydrides in microwave-assisted acylation, and in the liquid phase [20]. Phenolic substitution occurs with a high addition of succinic anhydride in reactive extrusion, and only aliphatic hydroxyl groups react with succinic anhydride in the liquid phase. The solubility of acetylated lignin prepared by supercritical carbon dioxide as a solvent is different from that which is prepared by the conventional liquid phase [16]. All acylated lignins that are prepared by microwave-assisted acylation with non-cyclic anhydrides as acylating agents are more thermally stable than the original lignin, and longer chains of anhydride lead to lower glass transition temperatures (*T*_g_) of acylated lignin [16,21]. The thermal stability of succinylated lignin prepared by the liquid phase method is better than the original lignin, but that which is prepared by reactive extrusion is worse [18,19].

Solid phase synthesis is an important green synthesis method for its simple process, environmental protection, low energy consumption, no deflagration, etc. However, it usually needs a long reaction time and it has a low reaction efficiency because the mixing and contact of reactants are inadequate, and the efficiency of the energy exchange is low. Mechanical activation (MA) is an effective method to enhance the contact and the interaction of reactants. It can change the structure of reactants, such as reducing the particle size, increasing the uniformity, and forming a new surface, thus improving the reactivity of reactants [22,23]. MA combined with solid phase synthesis can greatly improve the efficiency of the reaction, simplify the operation, and make full use of mechanical energy for the activation and reaction, which can be performed using the same equipment. In our previous works, the effect of MA on structure changes and reactivity in further chemical modification of sugarcane bagasse, cassava stillage residue, and lignin were studied [24,25,26]. Acetylated lignin, cellulose fatty acid esters with different chain lengths, and high-fatty acid starch esters were synthesized by MA-assisted solid phase synthesis (MASPS) [27,28,29]. MA increases the activity of hydroxyl groups in cellulose and lignin, and promotes the accessibility of reactants, thus enhancing the efficiency of esterification [30]. MASPS is a promising modification method for polymers, due to its low demand of equipment, its simple operation, and the easy scale of production.

Due to the reaction characteristics and product properties of lignin being affected by esterifying agents and preparation methods, in this study, acylated lignins with substituents of different carbon chain lengths were prepared by MASPS with non-cyclic and cyclic anhydrides as acylating agents. The effect of acylating agents on the reaction and the properties of the products were investigated in order to learn about the characteristics of MASPS in the modification of lignin.

## 2. Materials and Methods 

### 2.1. Materials

Lignin used in this study was same as that of reference [26]. All chemical reagents were of analytical grade and were obtained commercially without further purification.

### 2.2. Preparation of Acylated Lignins

The MA-assisted solid phase reactor was a customized stirring ball mill [31]. A fixed amount of zirconium oxide milling balls (300 mL, 5 mm diameter, ratio of milling balls to materials was 6 mL/g) was first added into a jacketed stainless steel chamber (1200 mL), and then a mixture of lignin, acylating agent, and catalyst (DMAP) (with lignin as reference, the dosages of anhydride and catalyst were 3 mol/ C_9_-units (benzenepropyl unit (C6～C3))and 2 wt %, respectively) was added into the chamber, and was subjected to milling at a fixed stirring speed of 300 r/min, and a constant temperature of 80 °C, controlled by circulating the thermostatic water in the jacket of chamber. When the mixture was milled for a desired length of time, the crude product was separated from the milling balls by a sieve. The resulting sample was washed and filtered repeatedly with deionized water (the product of succinylation was washed with anhydrous ethanol) until the filtrate was neutral. The filter cake was oven-dried at 50 °C for 24 h, and then the acylated lignin was obtained. The sample was sealed with a plastic bag and stored in a silica-gel desiccator.

### 2.3. Characterization

The structure features of lignin and its esters were determined by ultraviolet/visible (UV/Vis) spectroscopy (2802s UV/Vis spectrometer, UNIC, NJ, USA), Fourier transform infrared spectroscopy (FTIR) (FTIR -7600, Lambda Scientific Pty Ltd., Marion, Adelaide, Australia), and nuclear magnetic resonance (NMR) spectroscopy (AVANCE III HD 600 spectrometer, Bruck, Zurich, Switzerland),). The surface morphology of the samples was analyzed by scanning electron microscopy (SEM) with an S-3400N scanning electron microscope (Hitachi, Tokyo, Japan). The thermal properties of the original and the acylated lignins were determined by differential scanning calorimetry (DSC) (Q20 V24.4 Build 116, TA Instruments, New Castle, DE, USA) and thermal gravimetric analysis (TGA) (Q50 V20.10 Build 36, TA Instruments, New Castle, DE, USA). Detailed operating conditions of the characterizations are provided in the Supplementary Materials.

## 3. Results and Discussion

### 3.1. Effect of Acylating Agent on the Esterification of Lignin

Five anhydrides (acetic, propionic, butyric, maleic, and succinic anhydrides) were used as acylating agents in this study. When liquid anhydride was used, the system was a viscous paste at the beginning of the reaction, but the materials soon wrapped around the balls and became solid materials which stuck together with the balls. The impact and collision of the balls enhanced the reaction of the materials loading onto the surface. Therefore, the reaction system was mainly in the solid-state, despite it being slightly damp in the earlier stage. Although all the anhydrides except succinic anhydride were liquid substances, the use of anhydrides to study the characteristics of MASPS in the modification of lignin was meaningful.

#### 3.1.1. UV/Vis Analysis

In the UV/Vis analysis, the position and intensity of the maximum absorption related to the type of lignin, modification methods, solvents, etc. [32]. The types of phenolic hydroxyl groups could be determined for the different types of phenolic hydroxyl groups in lignin, corresponding to the different frequencies of ultraviolet light at the maximum absorption. The content of the phenolic hydroxyl groups was calculated by Equation (1) based on the Beer-Lambert law:

[OH]_λ_% = Δ*D*/Δε × 17 × 100% (1)

where [OH]_λ_% is the UV/Vis absorptivity of the phenolic hydroxyl groups at wavelengths of λ; Δ*D* is the specific difference absorptivity (Δ*D* = *D*_ionized_ − *D*_neutral_, *D*_ionized_ is the specific absorptivity of the sample in alkaline solution, and D_neutral_ is the specific absorptivity of the sample in neutral solution); *D* (L/(g·cm)) is the specific absorptivity (*D* = *A*/(*c* × *b*), *A* is the absorbance, *c* (g/L) is the concentration, and *b* (cm) is the light pathway (thickness of the cuvette’s wall)); Δε is the difference of molar absorptivity, and can be obtained according to the empirical Equation (2) as reported by Lin and Dence [32,33]:

Δε = 1.435 × 10^5^ – 4.15 × ν (2)

where ν is the wavenumber.

Chemical modifications that introduce unsaturated bonds into the side chain will cause a bathochromic shift of the absorption maxima, while the reaction that results in the blocking of phenolic hydroxyl groups or the reduction of chromophoric structures, will bring about hypsochromic and hypochromic changes in the spectrum. The extent of the modification can be determined according to these spectral changes [32]. Both phenolic hydroxyl and aliphatic hydroxyl groups in lignin could participate in esterification. Esterification of phenolic hydroxyl groups would result in a hypsochromic shift of the absorption maxima, due to the blocking of phenolic hydroxyl groups, while the esterification of aliphaticic hydroxyl groups would result in a bathochromic shift for the introduction of unsaturated carbonyl groups in the side chain. In addition, the esterification of not only phenolic hydroxyl groups, but also the aliphaticic hydroxyl groups, would lead to the decrease in the absorptivity of phenolic hydroxyl groups, for the latter would result in a decreased concentration of the phenolic hydroxyl groups due to the increased molecular weight from introducing the acyl groups.

**Figure 1:**
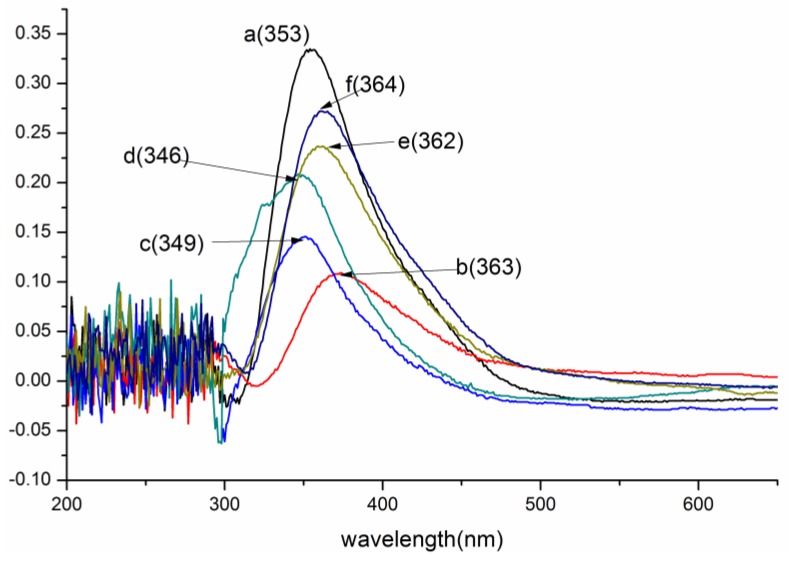
UV/Vis spectra of lignin and its esters: (**a**) original lignin; (**b**) acetylated lignin (1.5 h); (**c**) propionylated lignin (1.5 h); (**d**) butyrated lignin (2 h); (**e**) succinylated lignin (2 h); (**f**) maleylated lignin (2 h).

**Figure 2:**
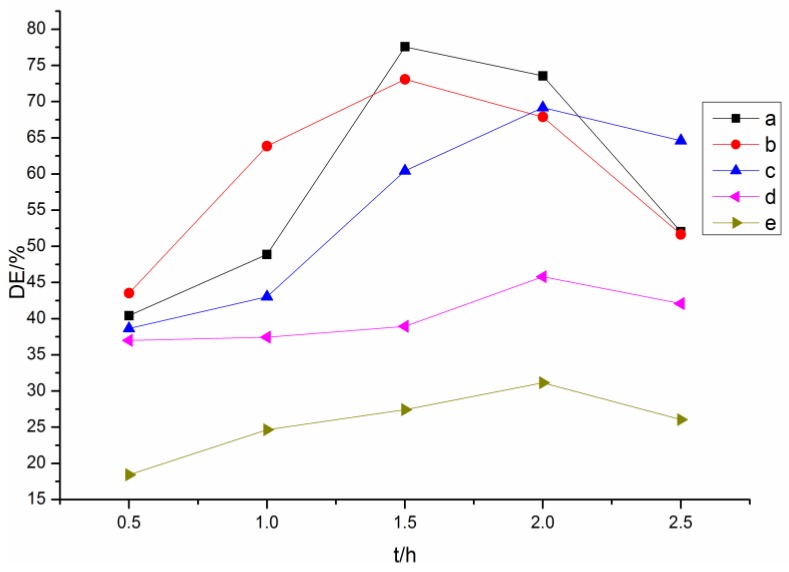
Degrees of esterification (Des) of lignin esters with different reaction times: (**a**) acetylated lignin; (**b**) propionylated lignin; (**c**) butyrated lignin; (**d**) succinylated lignin; (**e**) maleylated lignin.

Herein, ion differential spectrum analysis was used to determine the structure and phenolic hydroxyl groups content of the acylated lignins, and degree of esterification (DE) was calculated by comparing the content of phenolic hydroxyl groups before and after the esterification [10]. Ion differential spectra of the original lignin and its esters with an optimum reaction time, which corresponded to the best DE, are shown in Figure 1. DEs of the acylated lignins with different reaction times are shown in Figure 2.

The maximum absorption of the original lignin was around 350 nm, corresponding to the lignin of type 3 that was reported by Lin [32]. The absorptivity decreased significantly after esterification, illustrating that many hydroxyl groups participated in the reaction. For the lignin esters prepared with non-cyclic (acetic, propionic, butyric) anhydrides (Figure 1b–d and Figure 2a–c), the optimum reaction time for the acetylated and propionylated lignins was 1.5 h, while that for butyrated lignin was 2 h. The order of DE of the products with optimum reaction time was butyrated lignin < propionylated lignin < acetylated lignin, which indicated that the reactivity decreased with an increasing chain length of acyl groups because the space steric hindrance of the acyl groups increased with increasing chain length. The maximum absorption of acetylated lignin exhibited a bathochromic shift from 353 to 363 nm, indicating that aliphatic hydroxyl groups involved in the reaction were more than the phenolic hydroxyl groups. Meanwhile, the maximum absorptions of the butyrated and propionylated lignins exhibited hypsochromic shifts, showing that phenolic hydroxyl groups involved in the reaction were more than the aliphatic hydroxyl groups. Thus, both aliphatic hydroxyl and phenolic hydroxyl groups participated in the reaction during the acylation of lignin with non-cyclic anhydrides, and the reactivity of the phenolic hydroxyl groups were greater than that of the aliphatic hydroxyl groups. Therefore, the amount of the phenolic esters was more than that of aliphatic esters in propionylated and butyrated lignins for the low reactivity of the propionic and butyric anhydrides. However, the amount of the aliphatic esters was more than that of the phenolic esters in acetylated lignin for the higher reactivity of acetic anhydride, which was consistent with the content of the aliphatic hydroxyl groups, which was more than that of the phenolic hydroxyl groups in the original lignin.

For the esterified lignins prepared with cyclic (maleic, succinic) anhydrides (Figure 1e,f and Figure 2d,e), the DEs were far less than those of the esterified lignins prepared with non-cyclic anhydrides, and the optimum reaction time was 2 h. It could be due to this that the space steric hindrance made the formation of the ionized acyl groups difficult. Both these two maximum absorptions exhibited hypsochromic shifts, and the absorptivity of maleylated lignin was stronger than that of succinylated lignin. Thus, the aliphatic hydroxyl groups involved in the reaction were more than phenolic hydroxyl groups, which was consistent with reference [17], and the reactivity of maleic anhydride was slightly lower than that of succinic anhydride, due to the conjugation of double bonds and carbonyl groups. Whether the phenolic hydroxyl groups participated in the reaction was investigated by the following FTIR and NMR analyses.

#### 3.1.2. FTIR Analysis

**Figure 3:**
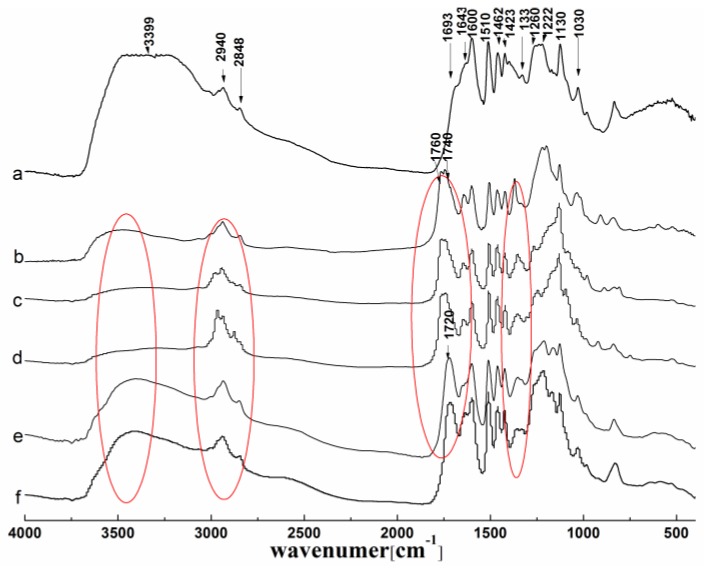
FTIR spectra of lignin and its esters: (**a**) original lignin; (**b**) acetylated lignin (1.5 h); (**c**) propionylated lignin (1.5 h); (**d**) butyrated lignin (2 h); (**e**) succinylated lignin (2 h); (**f**) maleylated lignin (2 h).

The FTIR spectra of the original lignin and its esters with the optimum reaction time are shown in Figure 3. For the original lignin, the peaks at 1600, 1510, 1460, and 1423 cm^−1^, corresponding to the aromatic ring skeleton, were distinguishable [16]. The peaks at 1260 cm^−1^ (C–H plane vibration of G unit), 1220 cm^−1^ (C–O–C of G-units), and 1330 cm^−1^ (C–O and C–C of S units) showed the characteristics of the G and S units in lignin [34].

Compared with the spectrum of original lignin, it is easy to observe from the spectra in Figure 3b–d that all of the acylated lignins were successfully prepared by MASPS. The peaks at 1760 and 1740 cm^−1^, corresponding to the carbonyl group of the phenolic esters and aliphatic esters respectively, appeared in the esterified lignins that were prepared with non-cyclic (acetic, propionic, butyric) anhydrides, so both the phenolic hydroxyl and aliphatic hydroxyl groups took part in the esterification. While there was only a peak at 1720 cm^−1^ which resulted from the overlapping of the carbonyl group of COOH (1695–1715 cm^−1^), and the carbonyl group of the aliphatic esters (1740 cm^−1^) appeared in the esterified lignins that were prepared with cyclic (maleic, succinic) anhydrides (Figure 3e,f), so only the aliphatic hydroxyl groups participated in the esterification. Therefore, the esterification reactivity of the hydroxyl groups in lignin with different acylating agents by MASPS was similar to the reaction by liquid phase synthesis [7].

In addition, there were three other remarkable differences in the spectra of the products, due to the reactivity and the structure of the acylating agents. For the acylated lignins prepared with non-cyclic (acetic, propionic, butyric) anhydrides, the absorptions of the whole hydroxyl groups (3300–3500 cm^−1^) reduced greatly, and the peaks of the phenolic hydroxyl groups (1300–1350 cm^−1^) became narrow, while the absorptions of the alkyl groups (2800–3000 cm^−1^) increased obviously, indicating the high efficiency of the esterification. For the acylated lignins prepared with cyclic (maleic, succinic) anhydrides, the peaks of the whole hydroxyl groups and the phenolic hydroxyl groups were broad, and the peaks of alkyl groups had no significant change, indicating the low efficiency of esterification. With a peak at 1600 cm^−1^ corresponding to the aromatic ring skeleton as a reference, the absorption of the whole hydroxyl groups had the following order: maleylated lignin > succinylated lignin > butyrated lignin > propionylated lignin > acetylated lignin. This indicated the reverse order of reactivity, which was consistent with the UV analysis.

#### 3.1.3. NMR Analysis

**Figure 4:**
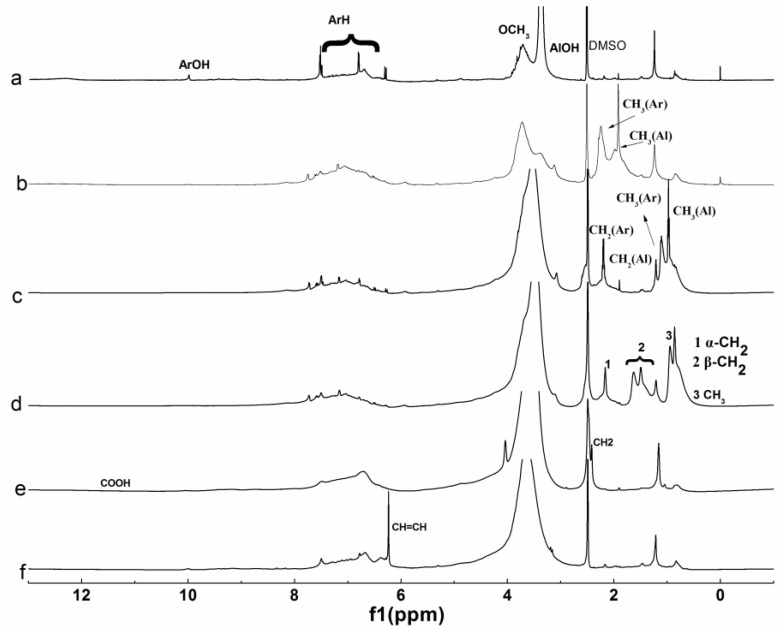
^1^H NMR spectra of lignin and its esters: (**a**) original lignin; (**b**) acetylated lignin (1.5 h); (**c**) propionylated lignin (1.5 h); (**d**) butyrated lignin (2 h); (**e**) succinylated lignin (2 h); (**f**) maleylated lignin (2 h).

**Figure 5:**
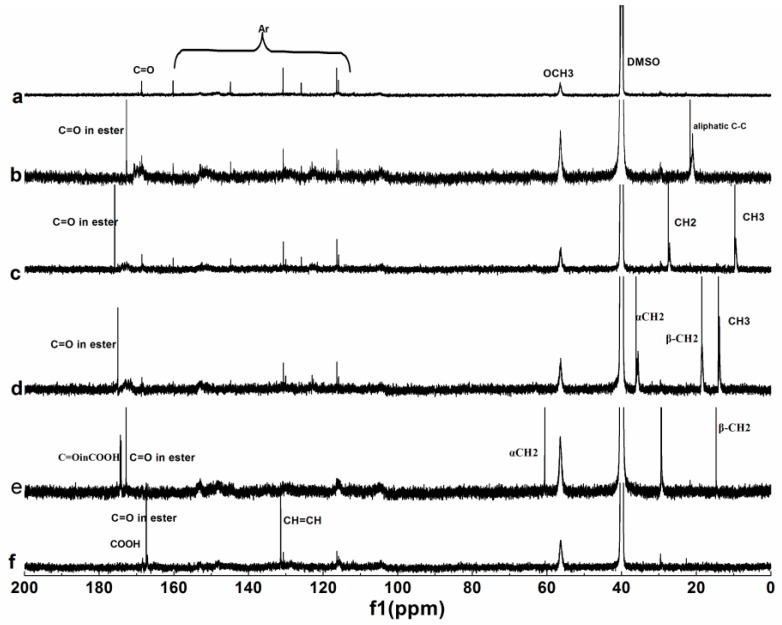
^13^C NMR spectra of lignin and its esters: (**a**) original lignin; (**b**) acetylated lignin (1.5 h); (**c**) propionylated lignin (1.5 h); (**d**) butyrated lignin (2 h); (**e**) succinylated lignin (2 h); (**f**) maleylated lignin (2 h).

^1^H and ^13^C NMR of the original lignin and its esters with the optimum reaction time were shown in Figure 4 and Figure 5, and the related attributions were also marked out. The spectra were similar to those prepared by liquid phase synthesis [7].

For the ^1^H NMR spectrum of original lignin, the peaks at 6–8, 3–3.5, 3.5–4, 8–10, and 1.7 ppm corresponding to the aromatic ring skeleton, aliphatic hydroxyl, methoxy, phenolic hydroxyl, and alkyl groups respectively, were distinguishable, which were typical characteristics of lignin [35]. The changes of the peaks at 1.5–2.6 ppm corresponding to alky groups were obvious after esterification, except maleylation. Both aliphatic hydroxyl and phenolic hydroxyl groups participated in the reaction, when the acylation of lignin were carried out with non-cyclic anhydrides, while only the aliphatic hydroxyl groups participated in the reaction when the acylation of lignin was carried out with cyclic anhydrides. For the ^13^C NMR spectrum of original lignin, the peaks at 110–150, 56, and 168 ppm corresponding to aromatic ring skeleton, methoxy, and carbonyl groups respectively, are obvious. The peaks at 0–70 and 160–178 ppm corresponding to alkyl and carbonyl groups changed obviously after esterification.

For the ^1^H NMR analysis, 6–8 ppm corresponding to aromatic hydrogen was chosen as the reference, the extent of the reaction was evaluated by the integral area of CH_3_ in Figure 4b–d, divided by 3, the integral area of CH_2_ in Figure 4e was divided by 4, the integral area of CH in Figure 4f was divided by 2, and the results were 0.540, 0.471, 0.452, 0.153, and 0.102, respectively. For the ^13^C NMR analysis, methoxy groups were chosen as the reference, and the extent of the reaction was evaluated by the integral area of the carbonyl groups of the ester in Figure 5b–d, the integral area of α–CH_2_ in Figure 5e, the integral area of CH=CH in Figure 5f was divided by 2, and the results were 0.613, 0.584, 0.450, 0.271, and 0.213, respectively. So, the extent of the reaction had the following order: b > c > d > e > f, which was consistent with UV and FTIR analyses.

Therefore, during the acylation of lignin by MASPS, both the aliphatic hydroxyl and the phenolic hydroxyl groups participated in the reaction when non-cyclic anhydrides were used as acylating agents, but only the aliphatic hydroxyl groups participated in the reaction when the cyclic anhydrides were used as acylating agents. The reactivity of the non-cyclic anhydrides was higher than that of the cyclic anhydrides, and the reactivity of acylating agent decreased with increasing chain length and the unsaturated bond of acyl groups.

### 3.2. Effect of Acylating Agent on Morphology and Thermal Properties of the Products

#### 3.2.1. SEM Analysis

**Figure 6:**
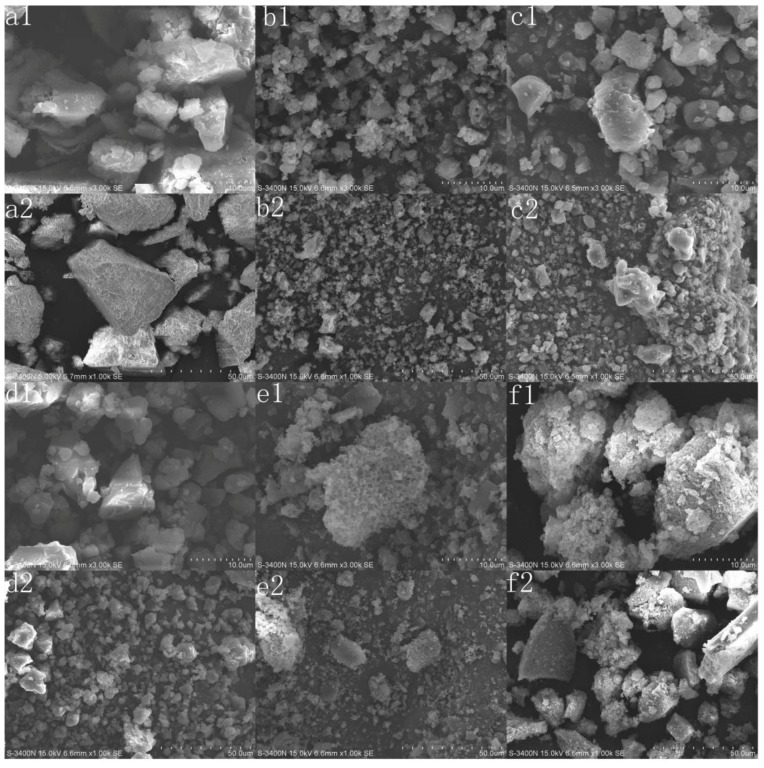
SEM analysis of lignin and its esters: (**a1**,**a2**) original lignin; (**b1**,**b2**) acetylated lignin (1.5 h), (**c1**,**c2**) propionylated lignin (1.5 h), (**d1**,**d2**) butyrated lignin (2 h), (**e1**,**e2**) succinylated lignin (2 h); (**f1**,**f2**) maleylated lignin (2 h).

The micrographs of original lignin and its esters with the optimum reaction times were analyzed by SEM, and the results are shown in Figure 6. The original lignin exhibited a large irregular block structure, with a compact interior and smooth surface. While the esters prepared with non-cyclic anhydrides exhibited a dispersed structure, with a loose interior and a rough surface. This could result from the acylation reaction consuming many hydroxyl groups and destroying the intermolecular hydrogen bonding. In addition, the particle size increased significantly with the increasing chain length of acylating agents for the decrease of DE. Although the consumption of hydroxyl groups and the destruction of intermolecular hydrogen bonding also occurred, the esters prepared with cyclic anhydrides still had large irregular block structures because the DEs were relatively small and new hydrogen bonds could be formed, due to the introduction of carboxyl groups. At the same time, the erosion of the acylating agent and the increase of side chain length led to the rough surface.

#### 3.2.2. DSC Analysis

**Figure 7:**
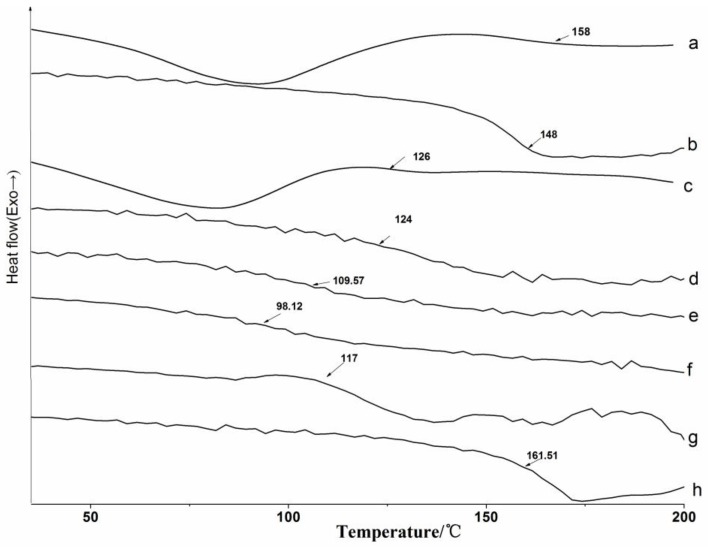
DSC analysis by direct measurement for (**a**) original lignin and (**c**) acetylated lignin (1.5 h); and measured by second heating for (**b**) original lignin; (**d**) acetylated lignin (1.5 h); (**e**) propionylated lignin (1.5 h); (**f**) butyrated lignin (2 h); (**g**) succinylated lignin (2 h); (**h**) maleylated lignin (2 h).

DSC analysis was adopted to measure the glass transition temperature (*T*_g_) of lignin and its esters, and the DSC thermograms are presented in Figure 7. Lignin and its esters have strong hydrophilicity for their abundant hydroxyl groups, so there were endothermic peaks at 80 °C for evaporation of water during the direct measurement (Figure 7a,c). In addition, the water also affected the *T*_g_ of lignin and its derivatives. The *T*_g_ measured by the direct scan were larger than that which was measured by second heating, which indicated that *T*_g_ was affected by the thermal history of the corresponding samples, and the influence on the original lignin was larger than on the acylated lignins. The difference of the direct scan and the second heating in this study was converse to the literature [4], and it could be attributed to the different annealing temperatures and times. The measurement was then carried out after eliminating the thermal history of samples (Figure 7b,d–i). All of the *T*_g_ values were in the region of 90–160 °C, which were slightly lower than those of the products that were prepared by the microwave-assisted method [16]. All of the acylated lignins, except maleylated lignin, had lower *T*_g_ than the original lignin. It was difficult to obtain fully esterified lignins or lignin esters with the same DEs. As a consequence, the more the lignin was acylated, the greater the decrease of *T*_g_ for the resulting lignin ester [14]. The long-chain lignin ester with lower DE still had a lower *T*_g_ than the short chain lignin ester, so that the *T*_g_ of acylated lignins prepared with non-cyclic anhydrides decreased with the increasing chain length of the acylating agents, which was consistent with the liquid phase and the microwave-assisted synthesis methods [16,21]. The *T*_g_ of the acylated lignins prepared with cyclic anhydrides was higher than that those with no cyclic anhydrides with the same carbon chain lengths. The maleylated lignin had no *T*_g_, but had a decomposition temperature (*T*_d_) at 161.5 °C due to the unsaturated bond, which could be proved by the following TG analysis.

#### 3.2.3. TG Analysis

**Figure 8:**
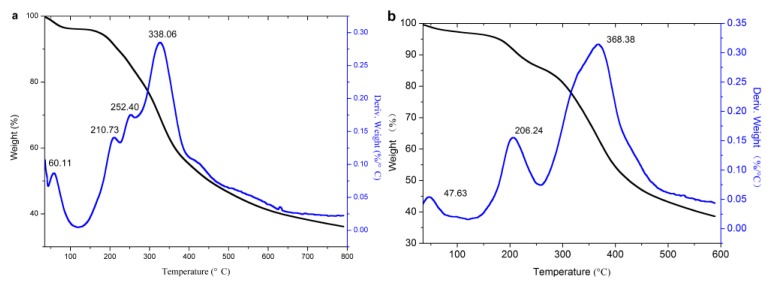

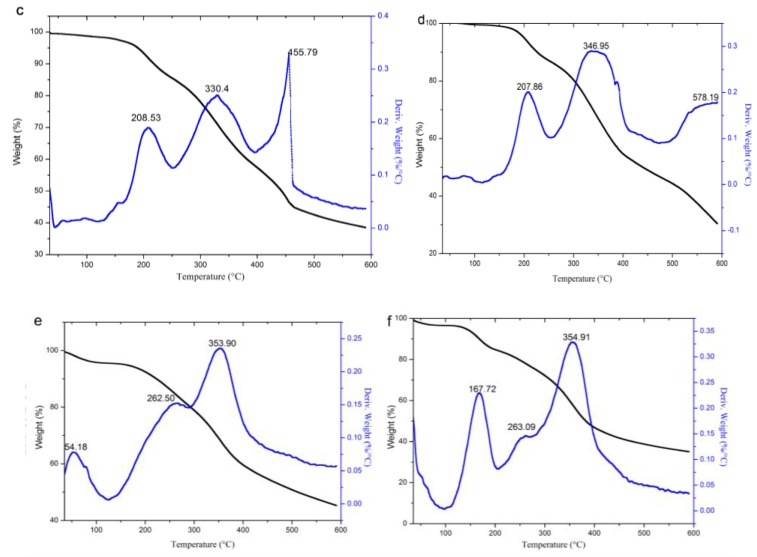
TG analysis of lignin and its esters: (**a**) original lignin; (**b**) acetylated lignin (1.5 h); (**c**) propionylated lignin (1.5 h); (**d**) butyrated lignin (2 h); (**e**) succinylated lignin (2 h); (**f**) maleylated lignin (2 h).

TG analysis was adopted to analyze the thermal stability of the original lignin and its esters, and the results are shown in Figure 8.

It can be seen from the DTG curves that the weightlessness of lignin and its esters were divided into three stages, and the related parameters are listed in Table 1. The first stage (30–125 °C) was the evaporation of water, which could be proved from DSC analysis in that it disappeared in the curves of the second heating. In this stage, the original lignin had an obvious peak, due to its strong hydrophilicity, because it contains a large number of hydroxyl groups. The acylated lignins prepared with non-cyclic anhydrides (except acetic anhydride) had nearly no peak, due to the weak hydrophilicity, because esterification consumed a large number of hydroxyl groups and introduced alkyl groups. The acetylated lignin still had an apparent peak for the improvement of hydrophilicity by short alkyl groups, but it was not as good as with the long alkyl groups. The acylated lignins prepared with cyclic anhydrides still had apparent peaks due to their strong hydrophilicity for the introduction of carboxyl groups and the consumption of hydroxyl groups occurred at the same time during the esterification. The second stage (125–268 °C) was caused by the degradation of the phenylpropanoid side chain of lignin [36]. In this stage, the original lignin had two peaks, while the acylated lignins except maleylated lignin had single peaks, which might have resulted from that the consumption of hydroxyl groups, which simplified the functional groups in the side chains of the lignin. Because of its unsaturated double bonds, maleylated lignin had a distinguishable decomposition peak around 167 °C. The third stage (268–600 °C) was the decomposition stage of the majority, during which the main units in the lignin esters were decomposed into small molecules, such as methane, ethane, propane, carbon monoxide, carbon dioxide, and hydrogen, etc. [4]. The propionylated and butyrated lignins had two peaks, which might be ascribed to the ester groups, and the main units had different stabilities due to the long chains of ester substituents. As acetyl groups were too short to have differences in stability from the main units, the acetylated lignin had a single peak. For succinylated and maleylated lignins, the ester substituents with carboxylic groups could form hydrogen bonding with the main units, and they easily formed a whole, so they still had a single peak.

It is hoped that the lignins are stable at high processing temperatures (e.g., 191–250 °C for polypropylene) when modified lignins are used as fillers in a thermoplastic matrix. Thus, the thermal stability in the first and second stages (Table 1) is important. For the acylated lignins prepared with non-cyclic anhydrides, the weight loss rate in first and second stages decreased obviously, and the temperature of the 5% weight loss rate and the maximum weight loss rate increased significantly, so their thermal stability was remarkably improved compared with the original lignin. In addition, the longer chains of acyl groups led to better thermal stability. For the acylated lignins prepared with cyclic anhydrides, their thermal stability was slightly improved, seen as only a minor improvement in the weight loss rate in the first stage and in the maximum weight loss rate. Thus, the introduction of carboxylic groups and unsaturated bonds in lignin reduce its thermal stability. It can be concluded that non-cyclic long chain anhydrides without carboxylic groups and unsaturated bonds are the ideal acylating agents for the esterification of lignin, in order to improve its thermal stability, while cyclic anhydrides are more suitable for preparing the precursors to lignin-based materials.

## 4. Conclusions

Acylated lignins with different carbon chain lengths in the ester substituents were successfully prepared by MASPS technology, with non-cyclic and cyclic anhydrides as acylating agents. Both aliphatic hydroxyl and phenolic hydroxyl groups participated in the acylation of lignin with non-cyclic anhydrides. The reactivity of acylating agents decreased with increasing chain length of acyl groups, which resulted in the different relative content of phenolic and aliphatic esters in the products. The reactivity of cyclic anhydrides was lower than that of the non-cyclic anhydrides, and only the aliphatic hydroxyl groups of lignin reacted with cyclic anhydrides in the acylation of lignin. The unsaturated bond in acylating agents also reduced their reactivities. The destruction of intermolecular hydrogen bonding by the esterification and erosion of acylating agents on lignin led to the loose interior and the rough surface of acylated lignins. All of the acylated lignins except maleylated lignin had lower *T*_g_ than original lignin. The acylated lignins that were prepared with non-cyclic anhydrides had better thermal stabilities than the original lignin, and the thermal stability increased, but *T*_g_ decreased significantly, with increasing acyl group chain lengths. The acylated lignins prepared with the cyclic anhydrides had higher *T*_g_ and worse thermal stability than those that were prepared with non-cyclic anhydrides with the same carbon chain lengths. The lignin esters prepared with different acylating agents possessed special properties, which could be used for different applications.

## Figures and Tables

**Table 1:** TGA characteristic parameters.

Sample	First Stage (30–125 °C)		Second Stage (125–268 °C)		Temperature of 5% Weight Loss(°C)	Third Stage (268–600 °C)		Temperature of Max Weight Loss (°C)
	Peak (°C)	Weight Loss (%)	Peak (°C)	Weight Loss (%)		Peak (°C)	Weight Loss (%)	
Original lignin	60.01	3.97	210.73, 252.40	17.68	172.69	338.06	61.63	338.06
Acetylated lignin (1.5 h)	47.63	3.06	206.24	14.58	178.87	368.38	61.58	368.38
Propionylated lignin (1.5 h)	--	1.75	208.53	14.40	189.9	330.4, 455.79	62.53	455.79
Butyrated lignin (2 h)	--	0.92	207.86	14.26	199.6	346.95, 578.19	69.53	346.95
Succinylated lignin (2 h)	54.18	4.77	262.50	16.27	157.21	353.9	61.47	353.9
Maleylated lignin (2 h)	35	4.51	167.72, 263.09	23.03	140.3	354.91	54.7	354.91

## Supplementary Materials

The following are available online at http://www.mdpi.com/2073-4360/10/8/907/s1.
