# Supplementary material for: Acylation of Lignin with Different Acylating Agents by Mechanical Activation-Assisted Solid Phase Synthesis: Preparation and Properties
Source: Polymers (Basel). 2018 Aug 12;10(8):907. doi: 10.3390/polym10080907 (PMC6403724; doi:10.3390/polym10080907)
Supplement: Supplementary file 1 [file polymers-10-00907-s001.pdf]

## Supporting Information

# Acylation of lignin with different acylating agents by mechanical activation-assisted solid phase synthesis: preparation and properties

**Xiaohong Zhao [1,2], Yanjuan Zhang [1,*], Mei Yang [1], Zuqiang Huang [1,*], Huayu Hu [1], Aimin Huang [1] and Zhenfei Feng [1]**

1 School of Chemistry and Chemical Engineering, Guangxi University, Nanning 530004, China; mshong_200@163.com (X.Z.); yangmei@gxu.edu.cn (M.Y.); yuhuahu@163.com (H.H.); ham2003cn@163.com (A.H.); myAfeila@163.com (Z.F.)

2 College of Materials and Environmental Engineering, Hezhou University, Hezhou 542899, China

* Correspondence: zhangyj@gxu.edu.cn (Y.Z.); huangzq@gxu.edu.cn (Z.H.); Tel.: +86-771-3233728 (Z.H.)

**Notes**

The authors declare no competing financial interest.

**Detailed Analytical Conditions**

**Ultraviolet/Visible (UV/Vis) Spectroscopy Analysis**

UV/Vis analysis was performed with a 2802s UV/Vis spectrometer (UNIC, USA) in the wavelength range of 200–600 nm, at a slit width of 1 nm, and at moderate scan velocity.

Approximately 0.010–0.015 g of sample was dissolved in 10 mL of water and 1,4-dioxane mixture (water/1,4-dioxane volume ratio=1:9). The neutral solution was obtained by diluting 2.0 mL of the solution to 50.0 mL with pH 6 phosphate buffer, while the alkaline solution was obtained by diluting 2.0 mL of the solution to 50.0 mL with pH 12 sodium borate buffer.

The difference spectra were directly determined by measuring the absorbance of the alkaline solution relative to the neutral solution [1].

**Fourier Transform Infrared Spectroscopy (FTIR) Analysis**

FTIR analysis was performed with a FTIR-7600 spectrometer (Lambda Scientific Pty Ltd., Australia). Mixed powders of sample (2.0 mg) and KBr (200.0 mg) were pressed into a disk for testing. The spectra were recorded with 32 scans in the frequency range of 4000–400 $cm^{-1}$ with a resolution of 1 $cm^{-1}$. In order to compare the obtained spectra, each spectrum was normalized with the intensity of the absorbance peak attributed to a characteristic band of the aromatic skeletal vibrations (A1600 $cm^{-1}$). The normalization and the baseline correction were processed as reported by Gilarranz et al. [2].

**Nuclear Magnetic Resonance (NMR) Analysis**

$^{1}H$ and $^{13}C$ NMR spectra were accumulated on an AVANCE III HD 600 spectrometer (Bruck, Switzerland).Approximate 100 mg and 300 mg of sample were dissolved in 1 mL of DMSO-d6 (99.9% deuterated, 0.05% tetramethylsilane) for $^{1}H$ and $^{13}C$ NMR analysis respectively, and left overnight for complete dissolution. The solution was filtered with a 0.45 μm microporous filter membrane before testing.

**Scanning Electron Microscopy (SEM) Analysis**

The samples were fixed on a sample bench using a double glue tape, and coated with gold to improve the conductivity. Micrographs were taken to observe the surface morphologies of different samples at 1,000× and 3,000× magnifications.

**Differential Scanning Calorimetry (DSC) Analysis**

DSC curves of the samples were examined by a DSC Q20 V24.4 Build 116 analyzer (TA Instruments, New Castle, DE, USA). The samples, with accurate weights of 5–10 mg, were placed and sealed onto an aluminum plate for analysis. Direct measurement was run from 30 to 200 °C at a scanning rate of 10 °C/min ramp under nitrogen. The measurement by second heating was run under nitrogen using the following program: (1) initial scan: heat ramp from 30 to 145 °C at 10°C /min, isothermal state at 145 °C during 30 min, cooling to 0 °C at 10°C /min, isothermal state at 0 °C during 10 min, and (2) $T_g$ measurement: heat ramp from 0 to 200 °C at 10°C/min. After the measurement, the instrument was cooled to 30 °C at 30 °C/min prior to the next sample.

**Thermogravimetric Analysis (TG)**

TG curves of the samples were examined by a TGA Q50 V20.10 Build 36 analyzer (TA Instruments, New Castle, DE, USA). Scans were run from 30 to 600 °C at a 10 °C/min ramp under nitrogen.

**References**

1. Nevárez, L.A.M.; Casarrubias, L.B.; Celzard, A.; Fierro, V.; Torres, V.; Davila, A.C.; Lubian, J.R.T.; Sánchez, G.G.; Sánchez, A.G.G. Biopolymer-based nanocomposites: effect of lignin acetylation in cellulose triacetate films. *Sci. Technol. Adv. Mater.* **2011**, 12, 45006.
2. Gilarranz, M.A.; Rodríguez, F.; Oliet, M.; García, J.; Alonso, V. Phenolic OH group estimation by FTIR and UV spectroscopy application to organosolv lignins. *J. Wood Chem. Technol.* **2001**, 21, 387–395.
